# Cognitive Behavioral Therapy and Acceptance and Commitment Therapy (CBT-ACT) vs. Standard Care After Critical Illness Due to COVID-19: Protocol for a Pilot Randomized Controlled Trial

**DOI:** 10.3389/fpsyt.2022.907215

**Published:** 2022-07-15

**Authors:** Anders Håkansson, Maria Cronhjort, Pernilla Lidin-Darlington, Gisela Lilja, Anna Nilsson, Anna Schandl, Hans Friberg

**Affiliations:** ^1^Faculty of Medicine, Lund University, Lund, Sweden; ^2^Clinical Research Unit, Department of Psychiatry, Malmø, Sweden; ^3^Department of Clinical Science and Education, Södersjukhuset Karolinska Institutet, Stockholm, Sweden; ^4^Södersjukhuset AB Hospital, Stockholm, Sweden; ^5^Skåne University Hospital, Malmö, Sweden

**Keywords:** COVID-19, SARS-CoV-2, post-covid syndrome, randomized controlled trial, acceptance and commitment therapy

## Abstract

**Background:**

Post-covid syndrome is an emerging condition involving a wide range of symptoms, including high rates of poor mental health. The diagnostic relevance and clinical severity of these symptoms are largely unknown, and evidence for treatment of post-covid mental health symptoms is lacking. This protocol describes a pilot randomized clinical trial, primarily aiming to assess feasibility, participant adherence and satisfaction in a novel phycho-therapeutic intervention on post-covid anxiety and depression symptoms ≥1 year after critically ill COVID-19. Whether the intervention may generate improvements in post-covid depression, anxiety, post-traumatic stress and health-related quality of life (HRQoL) will be addressed in a following larger trial.

**Methods:**

A multicenter, investigator-initiated randomized controlled trial (Clinical Trial Identifier number NCT05119608) including Intensive Care Unit (ICU)-treated COVID-19 survivors, who display symptoms of anxiety and/or depression at follow-up 12 months after hospitalization (Hospital Anxiety and Depression Scale ≥8 for depression or anxiety). Eligible individuals are referred to a psychiatrist for structured diagnostic assessment and inclusion in the trial. Participants will be randomized to either a 10-week cognitive behavioral therapy intervention with added acceptance and commitment therapy (CBT-ACT) or standard care (primary care referral). Primary study outcome measure is feasibility and patient adherence, defined as the proportion of participants who consent to randomization and remain in the study including follow-up. Secondary outcome measures include reduced symptoms in the HADS depression/anxiety subscales, post-traumatic symptoms, HRQoL and user satisfaction at 3 months after the intervention.

**Discussion:**

This protocol describes a pilot trial to assess feasibility and preliminary effects of a structured psycho-therapeutic intervention to ameliorate mental health in a population severely affected by COVID-19, where evidence for structured psycho-therapy is lacking.

## Introduction

### The COVID-19 Pandemic and Mental Health

The coronavirus disease, COVID-19, has had a devastating impact on public health, with persisting symptoms after the acute infection in a substantial number of patients. This is typically referred to as a post-covid syndrome, characterized by several symptoms affecting quality of life (1–5). Mental health symptoms are common in reports of a post-covid syndrome (4, 6). Studies have shown persistent symptoms of clinical depression in up to 40 percent at 1-6 months after COVID-19 in patients treated either in hospital or at home (7), whereas lower rates of depression (15 percent) also have been reported after hospitalization for COVID-19 (8).

Specific features of the COVID-19 disease itself have been suspected to cause mental health problems (9–12), in addition to possible causes emerging from societal changes during the pandemic (13, 14). Neuro-cognitive symptoms in COVID-19 have been associated with risk of post-covid depression and anxiety (15), and inflammatory parameters are suggested to have a role in neurological, cognitive and mental disorders in COVID-19 (7).

### Post-covid Mental Health Symptoms in Post-ICU COVID-19 Survivors

It has been reported that people surviving the acute phases of a severe COVID-19 condition requiring intensive care are at high risk of developing mental health symptoms.

In a large cohort of COVID-19 patients, severity of illness was associated with worse pulmonary function at 6-month follow-up, and anxiety and depression were significantly more common among the critically ill than among other less severely ill COVID-19 survivors (32 percent vs. 22–23 percent). Other common findings were fatigue, muscle weakness and sleeping disorders (2). Likewise, post-traumatic stress symptoms have been reported to be twice as high in ICU-treated COVID-19 survivors compared to hospital-treated non-ICU patients with COVID-19 (16). One study demonstrated that a substantial proportion of post-critically ill COVID-19 patients had mental health problems after a median of 5 months. In particular, depression, anxiety, and post-traumatic stress were common, occurring in 33–36 percent (17). 6-month prevalence of depression, mood or psychotic disorders was seen in 28 percent of ICU-treated COVID-19 patients in a recent study, compared to 24 and 25 percent of non-hospitalized, and non-ICU-treated hospitalized patients (18). Thus, although there is data pointing toward a narrow difference in mental health consequences between ICU-treated and other COVID-19 patients (18), there is still reason to believe that severity of disease in COVID-19 is associated with long-term mental health problems.

### Research Gaps in Clinical Significance and Treatment of Post-covid Mental Health Symptoms

There is a relatively high frequency of poor mental health in the post-covid syndrome, and particularly in patients surviving severe COVID-19 requiring intensive care. However, follow-up studies of post-covid syndrome cohorts have so far, with few exceptions, included symptom descriptions rather than diagnostic constructs (11), and there is a lack of studies assessing interventions. To our knowledge, the only intervention study of post-covid mental health problems to date, is from Mazza and co-workers (19), demonstrating a significant effect of antidepressant medications in post-COVID-19 patients, but the study had no control group and a short follow-up.

Given the complexity and novelty of the post-covid syndrome and the uncertainty about pathological mechanisms and the long-term course, there is an urgent need for structured diagnostic understanding and need to incorporate mental health issues in structured follow-up and treatment of the condition (20). This includes development of structured diagnostic and interventional efforts (6) for post-covid mental health problems. Successful treatment of mental health symptoms in COVID-19 survivors is likely to require an understanding of the background of these symptoms and of the complexity of mental health suffering in combination with a severe physical condition. Acceptance and commitment therapy (ACT), originating from a cognitive behavioral therapy (CBT)-oriented framework, has been suggested as an intervention for other conditions where mental health symptoms develop in the context of a physical disorder. CBT/ACT (21) is well-suited for psychotherapy in situations where mental health problems occur in the context of a physical disease, or other complex conditions. This type of psycho-therapy is less commonly provided than standard CBT, but it is well-established and is part of the overall umbrella of interventions based on the core features of CBT, particularly in the context of specific, more complex conditions. This includes treatment of chronic pain (21), advanced malignant disease (22), or fibromyalgia (23). Likewise, the method has been suggested for the promotion of healthy lifestyle interventions (24) and occupational rehabilitation in patients on sick leave (25).

An additional research gap is whether a psycho-therapeutic treatment approach is feasible and accepted by patients with a history of critical illness due to COVID-19 infection. Thus, there is reason to investigate feasibility including adherence as well as user satisfaction with a psycho-therapeutic treatment initiated within the multi-disciplinary framework of COVID-19 rehabilitation and follow-up. The purpose of the present study is to assess long-term mental health symptoms in individuals who have survived critical COVID-19 requiring intensive care, and to scientifically test the feasibility of an integrated and adapted psycho-therapeutic intervention against mental health symptoms.

### Specific Aims

The present study aims to

a) Examine whether Acceptance and Commitment Therapy, based on the base framework of Cognitive Behavioral Therapy (CBT-ACT), compared to standard clinical care (referral to primary care), is feasible in critically ill COVID-19 survivors at12 months after ICU care, assessed by consent rate, recruitment success and protocol adherence (retention and successful follow-up rates).
b) Explore the diagnostic entities and clinical significance of the mental health symptoms reported in ICU-treated COVID-19 survivors at 12 months after ICU care.
c) Evaluate whether Cognitive Behavioral Therapy with Acceptance and Commitment Therapy (CBT-ACT), compared to standard clinical care (referral to primary care) has beneficial effects for amelioration of depression, anxiety, post-traumatic symptoms, fatigue, and for improvements of quality of life.

## Methods

### Study Population and Setting

The present study will include individuals after intensive care, in two Swedish settings; four ICUs in the Skåne Region (southern Sweden) and two ICUs at Södersjukhuset (Region Stockholm). Surviving individuals admitted to ICU-care are invited to a structured follow-up after 12 months, including self-reported questionnaires regarding mental health problems. The 12-month follow-up measures are carried out through physical (or telephone) follow-up visits (Region Skåne) or through questionnaires sent by regular mail (Stockholm study site). COVID-19 survivors who screen positively for clinical anxiety or depression, defined as a score of ≥8 on either of the two sub-scales within the questionnaire HADS (26), are invited to take part in the study. Eligible individuals will then undergo a structured online clinical diagnostic assessment by a physician experienced in clinical psychiatry. The structured psychiatric medical assessment is guided by the Mini Neuropsychiatric Interview [MINI, (27)]. The MINI interview provides preliminary, structured diagnoses according to the diagnostic manual of the American Psychiatric Association, the DSM-5 [American Psychiatric Association, (28)]. The sections chosen for this assessment include those related to major depression, generalized anxiety disorder, and post-traumatic stress. In addition to the abbreviated MINI assessment, a non-structured clinical interview and broad diagnostic assessment will be performed.

After the assessment, eligible ICU survivors will be offered to enter the study. Consenting participants will be randomized on an equal basis (1:1) to the intervention or control arm. Randomization will be carried out from concealed, opaque envelopes, containing randomization or control assignment as based on a computer-generated randomization list [random.org, (29)], handled by a secretary not involved in the research project. Exclusion criteria, assessed upon the medical examination, include conditions in which participants are acutely suicidal, psychotic, suffering from a mental disorder requiring in-patient psychiatric treatment, opposed to receiving a psycho-therapeutic intervention, or with cognitive problems or language difficulties which are judged to be too extensive for the informed consent procedure and for a psycho-therapeutic intervention.

### The Intervention

A structured, 10-session psycho-therapeutic intervention, typically occurring once weekly, will be performed (with a possibility to adjust the frequency). The therapy is based on cognitive behavioral therapy (CBT), with the addition of a therapeutic model of acceptance and commitment therapy (ACT) provided by clinical therapists with standard CBT training and extensive experience of complex mental health disorders. The intervention will be provided face-to-face whenever possible, with an alternative to offer the intervention online in case of practical difficulties or circumstances making an online meeting more convenient for the participant.

The control group (comparator) will consist of a referral to primary care (general practitioner) for medical assessment and support. The control condition is expected to provide at least the same level of support and care as in everyday, non-research-related, clinical care.

### Outcomes

The primary outcome is to test if the psycho-therapeutic treatment approach is feasible in critically ill COVID-19 survivors at 12 months after ICU care, defined as the proportion of participants who consent to randomization and remain in the study including follow-up. Secondary outcome measures include reduced symptom burden in the HADS depression and anxiety subscales, post-traumatic symptoms and mental health-related quality of life (HRQoL), as well as users' experience and user satisfaction. Secondary outcome measures (Table 1) are included both in the 12-month assessment prior to randomization, as well as in repeated assessments at 3 months after randomization (2 weeks after treatment ending in the intervention group), 3 months after the completion of the intervention, and in a longer perspective (12 months after intervention/control), administered by an as outcome assessor blinded to the intervention arm. Participants' experience and satisfaction of the CBT-ACT intervention will be assessed 2 weeks after the intervention (for the randomized intervention group only).

**Table 1:** Outcome assessments at baseline and follow-up.

**Outcome**	**Outcome assessment**	**Baseline variables**	**Study population 12 months post-ICU-care**	**Follow-up 2 weeks after intervention**	**Follow-up 3 months after intervention**	**Follow-up 12 months after intervention**
Mental health	Hospital Anxiety and Depression Scale (HADS)		X	X	X	X
	Post-Traumatic Stress Disorders Checklist for DSM-5 (PCL-5)		X	X	X	X
Fatigue	Modified Fatigue Impact Scale (MFIS)		X	X	X	X
Health related Quality of Life	SF-36-v2 (full scale and mental health sub-scale)		X	X	X	X
Participants' satisfaction	Satisfaction with Therapy and Therapist Scale (STTS)			X (intervention group only)		
Demographic background data	Age, gender	X				
Clinical baseline information	Severity of disease on arrival (Simplified Acute Physiology Score3, SAPS3)	X				
	Progression of disease severity in the ICU (30) including Sequential Organ Failure Assessment (SOFA)	X				
	Severity of ARDS on admission and daily,	X				
	Time (days) in the ICU	X				
	Invasive/non-invasive mechanical ventilation (yes/no)	X				
	Time on ventilator (days)	X				
Self-reported psychiatric information	Previous mental health disorders		X			

### Study Variables

Depression and anxiety will be measured using the HADS, a well-established instrument for the assessment of depressive and anxiety symptoms in clinical settings, and has demonstrated satisfactory sensitivity, specificity and validity (26). Mental health-related quality of life (HRQoL) measured as improved scores in the mental component summary score (MCS) from the SF-36v2 at 3 months after the intervention. The SF-36v2 has proven satisfactory psychometric characteristics in Sweden (31) and elsewhere (32). Post-traumatic symptoms will be assessed by the Posttraumatic Stress Disorder Checklist (PCL-5). The PCL-5 instrument has demonstrated satisfactory internal consistency and validity measures in the measure of post-traumatic symptoms (33). Fatigue will be studied using the Modified Fatigue Impact Scale (34), an abbreviated version of the longer, previous Fatigue Impact Scale, with demonstrated satisfactory validity and internal consistency (35). PCL-5 and MFIS will be assessed at 3 months only for participants who score positive at baseline on post-traumatic stress, and fatigue, respectively. User experience and satisfaction, in the intervention group only, will be assessed with the Therapy and Therapist Scale-Revised (STTS-R), a scale assessing both the aspects of the therapy method and the satisfaction with the therapist, and which has proved to have good measures of internal consistency and validity (36).

### Data Analysis and Statistics

In addition to the comparison of the intervention and control conditions, respectively; multi-variable logistic regression model will be used to evaluate the associations between disease severity and socio-demographic variables and outcome measures. Study outcomes will be statistically controlled for a limited number of potentially confounding factors, including the presence of psychoactive medication in the participants' history, age, sex, and whether mechanical ventilation was applied during ICU treatment or not.

As the study includes feasibility as an outcome measure, and as the potential study group may expand in the future, the study sample is yet undetermined. For the feasibility measures, a preliminary aim is to include a convenience sample of at least 40 participants.

All analyses will be performed with an intention-to-treat approach for the total group and stratified for gender and age, since normative data suggest that females have more mental health problems than males, while older individuals have less problems than younger ones.

### Patient and Public Involvement

Patient organizations have not been directly involved in the design of the present trial. While this is a study protocol, the dissemination and implications of the study results will involve patient and peer-support organizations and public policy makers.

## Discussion

Treatment of mental post-covid syndrome is sparsely addressed in the literature. The demonstration of feasibility for the CBT-ACT intervention in the present study will have potential implications for treatment of many patients with the post-covid syndrome.

### Strengths and Limitations

This project can contribute to improved scientific hypotheses and will provide the base for a future, larger and adequately powered interventional trial in the present research group, as well as in other settings, addressing post-covid mental health symptoms with psycho-therapeutic interventions. The present study thus aims to address feasibility, adherence and participant satisfaction, i.e., to examine how survivors of severe COVID-19 perceive a CBT/ACT intervention which will be tested for the first time. This will add to the current knowledge and be of value in future academic and clinical work.

## Conclusions

This protocol describes a multicenter, randomized, assessor-blinded clinical pilot trial in patients with post-covid mental health problems at 12 months after ICU care. We hope that this pilot trial will be an important contribution to a structured psychiatric assessment of patients with a long-term course of post-covid and strengthen the rationale for future, adequately powered randomized multicenter trials. The addition of evidence for a therapeutic model for critically ill COVID-19 patients with persisting post-covid mental health problems would be beneficial for many patients and for society, and results will be of importance to the design of a larger, fully powered intervention study.

## Ethics Statement

The study was approved by the Swedish Ethics Review Authority (file number 2021/05128, approval date November 3, 2021, and amendments with file number 2021-06977-02 and approval date January 10, 2022, and with file number 2021-01277-02 and approval date March 14, 2022). The study was pre-registered on clinicaltrials.gov (Clinical Trial Identifier number NCT05119608). Feasibility data and early outcome data will give crucial information for future trials on the efficacy of a CBT-ACT intervention in individuals who suffer from long-term anxiety and depression.

## Author Contributions

AH wrote the first draft of the manuscript. All authors contributed substantially to the overall research idea and the practical and theoretical research planning, editing of the manuscript, and approved the final version of the manuscript and its submission.

## Funding

Swedish Heart and Lung Foundation (grant number: N/A). Regional funding Region Skåne and Region Stockholm, ‘ALF’ organization of Swedish health care research funding (grant number: N/A).

## Conflict of Interest

The authors declare that the research was conducted in the absence of any commercial or financial relationships that could be construed as a potential conflict of interest.

## Publisher's Note

All claims expressed in this article are solely those of the authors and do not necessarily represent those of their affiliated organizations, or those of the publisher, the editors and the reviewers. Any product that may be evaluated in this article, or claim that may be made by its manufacturer, is not guaranteed or endorsed by the publisher.
